# The Trunk Appearance Perception Scale (TAPS): a new tool to evaluate subjective impression of trunk deformity in patients with idiopathic scoliosis

**DOI:** 10.1186/1748-7161-7-S1-O1

**Published:** 2012-01-27

**Authors:** J Sánchez Raya, J Bagó, FJ Sánchez Perez-Grueso, JM Climent

**Affiliations:** 1 Hospital de la Vall d’ Hebron, Barcelona, Spain; 2 Hospital de La Paz, Madrid, Spain; 3 Hospital General de Alicante, Spain

## Background

Outcome assessment in idiopathic scoliosis should probably include patients’ perception of their trunk deformity in addition to self-image. This can be accomplished with the Walter Reed Visual Assessment Scale (WRVAS) [1-4]. Nevertheless, this instrument has some shortcomings. These considerations prompted us to design the Trunk Appearance Perception Scale (TAPS).

## Material and methods

Patients with idiopathic scoliosis and no prior surgical treatment were included. Each patient completed the TAPS and SRS-22 questionnaire and underwent a complete radiographic study of the spine [5,6]. The magnitude of the upper thoracic, main thoracic, and thoracolumbar/lumbar structural curves was recorded. The TAPS includes 3 sets of figures that depict the trunk from 3 viewpoints: looking toward the back, looking toward the head with the patient bending over and looking toward the front. Drawings are scored from 1 (greatest deformity) to 5 (smallest deformity), and a mean score is obtained.

## Results

A total of 186 patients (86% females), with a mean age of 17.8 years participated. The mean of the largest curve (CMAX) was 40.2°. The median of TAPS sum score was 3.6. The floor effect was 1.6% and ceiling effect 3.8%. Cronbach’s alpha coefficient was 0.89; the ICC for the mean sum score was 0.92. Correlation coefficient of the TAPS mean sum and CMAX was -0.55 (P < 0.01). Correlation coefficients between TAPS mean sum score and SRS-22 scales were all statistically significant, ranging from 0.45 to 0.52 (P < 0.05).

## Conclusions

The TAPS is a valid instrument for evaluating the perception patients have of their trunk deformity.
